# Reliability, Validity, and Gender Invariance of the Exercise Benefits/Barriers Scale: An Emerging Evidence for a More Concise Research Tool

**DOI:** 10.3390/ijerph18073516

**Published:** 2021-03-29

**Authors:** Stefan Koehn, Farzad Amirabdollahian

**Affiliations:** School of Health Sciences, Liverpool Hope University, Liverpool L16 9JD, UK; koehns@hope.ac.uk

**Keywords:** exercise benefits, exercise barriers, questionnaire, validity, reliability, confirmatory factor analysis

## Abstract

The Exercise Benefits/Barriers Scale (EBBS) research instrument has been extensively used to investigate the perceived benefits and barriers of exercise in a range of settings. In order to examine theoretical contentions and translate the findings, it is imperative to implement measurement tools that operationalize the constructs in an accurate and reliable way. The original validation of the EBBS proposed a nine-factor structure for the research tool, examined the EBBS factor structure, and suggested that various factors are important for the testing of the perception of exercise benefits and barriers, whereas a few items and factors may not be vital. The current study conducted a confirmatory factor analysis (CFA) using hierarchical testing in 565 participants from the northwest region of the United Kingdom, the results of which provided evidence for a four-factor structure of the benefits measure, with the Comparative Fit Index (CFI) = 0.943, Tucker–Lewis Index (TLI) = 0.933, and root means square error of approximation (RMSEA) = 0.051, namely life enhancement, physical performance, psychological outlook, and social interaction, as well as a two-factor structure of the barrier measures, with the CFI = 0.953, TLI = 0.931, and RMSEA = 0.063, including exercise milieu and time expenditure. Our findings showed that for a six-factor correlated model, the CFI = 0.930, TLI = 0.919, and RMSEA = 0.046. The multi-group CFA provided support for gender invariance. The results indicated that after three decades of the original validation of the EBBS, many of the core factors and items are still relevant for the assessment of higher-order factors; however, the 26-item concise tool proposed in the current study displays a better parsimony in comparison with the original 43-item questionnaire. Overall, the current study provides support for a reliable, cross-culturally valid EBBS within the UK adult population, however, it proposes a shorter and more concise version compared with the original tool, and gives direction for future research to focus on the content validity for assessing the perception of the barriers to physical activity.

## 1. Introduction

The health benefits of physical activity (PA), especially for the prevention of non-communicable diseases, and the recommendations for minimum PA to secure such health benefits have been well-documented [1]. Despite this, approximately 3.2 million deaths per year have been attributed to a lack of engagement in PA, and this has established physical inactivity as the fourth universal risk factor of mortality [1]. Thus, it is imperative to understand the barriers and facilitators of engagement in PA. Although obtaining such an understanding does not necessarily lead to enhancement of engagement in PA, such understanding is essential for providing more in-depth knowledge about the adherence to PA guidelines, the etiology of physical inactivity, and developing policies and strategies for facilitation of PA behavior [2].

Amongst several theories aiming to explain human health behavior, the Health Belief Model is one of the most widely-used applied theories [3], postulating that risk susceptibility, risk severity, self-efficacy, and cues to action, together with perceived benefits of action and perceived barriers of action, are the six key constructs predicting health behavior [4]. Among these constructs, perceived barriers and benefits are of particular importance, as it was previously demonstrated that the ratio of perceived barriers to perceived benefits is the strong predictor of the individual’s health [5] and PA [6] behavior. Previous studies in older adults demonstrated that the perception of the PA benefits can take intrinsic orientation represented in perspectives such as health promotion, self-confidence, and well-being; or extrinsic orientation via improving social interactions, receiving financial rewards, or attendance in a convenient environment [7]. Similarly, the perception of barriers of the PA is equally as important as actual or imagined barriers, inconveniences, predicaments, financial constraints, and the range of expenses linked with adherence and childcare, together with the perceived risk of safety and security have been previously reported as barriers adversely affecting PA behavior [8].

Previous studies have identified that, amongst adults, physical limitations due to pain and weakness, a lack of motivation, and a lack of time are the strongest perceived barriers to PA; whereas family relationships, social support, and potential health benefits are the strongest motivators [9]. In young adults, where external barriers and health education demonstrated a strong association with PA behavior [10], a study demonstrated that the availability of places to exercise and the convenience of the exercise schedule, together with physical exertion (hard work, tiredness, and fatigue) from exercise, were the strongest perceived PA barriers [11]. Amongst older adults, the review of Schutzer and Graves (2004) classified poor health, availability of a physical environment, a lack of advice from the physician, and the lack of knowledge and understanding of the relationship between PA and health, together with the pattern of insufficient PA during childhood and youth, as the established constraints to PA, while an individual’s belief in their ability to successfully perform (i.e., self-efficacy), continuous contacts and prompts, and environmental factors, such as appropriate music, were classified as the motivators to exercise [12]. Despite these broad themes, the determinant of PA behavior and perceptions of barriers and facilitators of such behavior are population-specific, based on biological, psychological, social, and cultural characteristics of the population [2], and thus, there is a need for a validated tool that can identify and examine the perceived barriers and facilitators of PA in different populations. Table 1 demonstrates a summary of the literature on the perception of the benefits and barriers of physical activity with a methodology and population comparable to the current study.

In the UK, El Ansari and Lovell [13] and Lovell, El Ansari, and Parker [6] investigated the barriers to exercise between non-exercising younger and older female adults using the barriers subscale of the Exercise Benefits/Barriers Scale (EBBS) in relation with family relationships and responsibilities; however, a relatively small sample size, the focus on barriers (and, in particular, in relation with the number of children), inadequate attention to confounding factors, narrow age and gender categories of the participants, and, most importantly, the descriptive analysis of the findings limited the generalizability and usefulness of the findings.

The EBBS research instrument, as employed by El-Ansari and Lovell [13], has been extensively used to investigate the perceived benefits and barriers of exercise in a range of settings, populations, and conditions, for instance in Mexican-American women [14], midlife Australian women [15], older African women [16], Iranian women [17], in physical disabilities and chronic health conditions [18], in patients with multiple sclerosis [19] or with HIV [20], in overweight and obese women with polycystic ovarian syndrome and as part of a randomized controlled trial [21], as part of the investigation into physiological and perceptual responses to Latin partnered social dance [22], in relation with cardiac rehabilitation [23,24], in parents and preschool age children [25,26], and as part of the investigation of the perception of a yoga-based fall prevention program in older adults [27]. The above studies have implemented the EBBS as part of the research, making reference to the original validation study by Sechrist, Walker, and Pender in 1987 [28].

The original validation of the EBBS, conducted by Sechrist and colleagues [28] in the USA, used a large sample of 664 adults (mean age = 38.7 years) and exploratory factor analysis (EFA) with varimax rotation to assess the factor structure. The results provided support for a 43-item and nine-factor solution, with five factors composing the benefits subscale and four factors the barriers subscale. Although no item cross-loadings were found between benefit and barrier subscales or within the barrier subscale, a number of items cross-loaded within the benefits subscale. The item factor loadings for all factors were acceptable, and ranged between 0.46 and 0.85. Factor loadings for first-order factors varied between 0.56 and 0.77. The nine-factor solution explained 64.9% of the variance. In addition, Cronbach’s alpha provided support for the reliability of the EBBS.

Following the validation study by Sechrest and colleagues [28], the EBBS nine-factor structure has been assessed on various occasions. In contrast to the original validation study, the subsequent factor analytical results only partially confirmed the original structure. The EFA assessment of Akbari Kamrani et al. (2014) of the EBBS accounted for 61.8% of the variance [29]. Although the amount of variance explained was similar to the Sechrest et al. study [28], in a sample with 388 older participants (above 60 years of age), Akbari Kamrani et al. (2014) found a 10-factor solution, and two items showed cross-loadings with other factors, yielding five benefit and five barrier factors [29]. Similarly, within the study of Brown (2005), including 398 psychology students, the use of EFA also yielded a 10-factor solution [30]. Interpretation of the factor structure was based on a factor loading cutoff of 0.45 and factors with less than three items; for instance, family discouragement with two items was omitted. Overall, a total of 17 items did not load on any factor, with loading below 0.45. The final seven-factor solution supported five benefit factors and two barrier factors, explaining 38.1% of the variance [30]. Using a sample of 409 nursing students, EFA assessment of the EBBS conducted by Ortabag et al. [31] also supported a seven-factor structure, with five benefit and two barrier factors, accounting for 57.1% of the variance. In sum, the original 43-item, nine-factor structure as proposed by Sechrest et al. [28] was only partially supported by follow-up studies. Thus, based on EFA results, it appears that the EBBS might be effective with fewer items and fewer factors. Benefit factors that have been confirmed repeatedly include physical performance, psychological outlook, social interaction, and preventive health, whereas barrier factors were reflected by physical exertion and exercise milieu [29,30,31].

The inconsistent findings discussed demonstrate a gap in the knowledge, and the fact that, despite the frequent use of EFA and substantial support for part of the EBBS factor structure, a methodological shortcoming in the psychometric assessment is the lack of rigorous confirmatory factor analysis of the EBBS.

The present study conducts a confirmatory factor analysis as part of its study of validity, and within the domain of construct validity. This conceptually refers to the extent that the scores of the EBBS are consistent with the hypotheses that they were generated for (and based on the presumption that the EBBS indeed validly measures the construct to be measured). The fundamental perspectives within this domain of validity are the structural validity, hypotheses validity, and cross-cultural validity [32]. Through incorporating CFA as part of our broad analysis, the study not only attempts to lessen the overall number of observations into latent factors based on the commonalities observed in the data, but also (and more importantly) to minimize measurement errors and facilitate inferential analysis and comparison of the alternatively postulated a priori model [33]. CFA therefore offers an opportunity to statistically examine the structure of groups within our target population, facilitate the investigation of the cross-cultural validity, and give adequate confidence for confirmation of the validity of the instrument and its sub-domains, or potentially developing an amended version of the research tool, if necessary.

The overall purpose of the current study was to examine the EBBS reliability, validity, and gender invariance. To meet this broad goal, we produced three aims linked with elements of the study.

Reliability tests of Cronbach’s alpha for the global EBBS and EBBS subscales have been previously tested [28,29,30,31], showing values above the 0.70 cutoff [39]. The first aim of the study was to re-test the reliability of the EBBS and underlying factors for our target population (Aim 1). Previous studies testing the EBBS factor structure [28,30,31] indicated that various factors are essential for the testing of exercise benefits and barriers, whereas a number of items and factors are not vital. In contrast to previous EFA approaches, we intended to assess the latent factor structure of the EBBS using CFA (Aim 2). An important aspect when testing exercise benefits and barriers is the potential moderating effect of gender. A methodological shortcoming of previous research was the testing of gender differences based on mean differences, such as *t*-tests [29]. A thorough investigation of gender differences would be the assessment of gender invariance based on covariance matrices. Therefore, we intended to examine the potential gender differences between exercise factors and item understanding using multi-group confirmatory analysis (Aim 3).

## 2. Materials and Methods

### 2.1. Participants

A total of 565 males (*n* = 244) and females (*n* = 321) between 15 and 41 years of age (M = 21.19, SD = 2.42) participated in this study. Data were collected from university students from Merseyside, UK. Most participants reported being single (*n* = 454; in a relationship, *n* = 101) at the time of data collection. For more information about the study population and the recruitment procedure, please see our earlier publications [40,41].

### 2.2. Instrument

The Exercise Benefits/Barriers Scale (EBBS; Sechrist et al., (1987)) was developed to assess people’s perceptions on exercise benefits and barriers to exercise. Sechrest et al. (1987) developed the EBBS with nine factors and 43 items, 29 items assessing exercise benefits and 14 items exercise barriers [28]. The EFA at the time revealed five factors underlying the benefits of exercise, which were labelled life enhancement (seven items in total; item example: “Exercise improves the quality of my work”), physical performance (nine items in total; item example: “Exercise increases my level of physical fitness”), psychological outlook (six items in total; item example: “I enjoy exercise”), social interaction (four items in total; item example: “Exercising is a good way for me to meet new people”), and preventive health (three items in total; item example: “I will live longer if I exercise”). Four factors depict barriers to exercise, termed as exercise milieu (six items in total; item example: “It costs too much to exercise”), time expenditure (three items in total; item example: “Exercising takes too much of my time”), physical exertion (three items in total; item example: “Exercise is hard work for me”), and family discouragement (two items in total; item example: “My family members do not encourage me to exercise”). The response scale is a four-point Likert-type scale anchored by 1 (strongly agree) and 4 (strongly disagree).

### 2.3. Procedures

Following approval by the university’s ethics committee, the study was promoted across the universities in Merseyside, UK. The current investigation is part of a large cross-sectional study, where participants were recruited and attended two clinical visits for physical and physiological assessments in the first visit, and demographic and questionnaire completion within the second visit. Standard consent procedures were followed before questionnaire administration. Participants filled in the demographic questionnaire and the EBBS, together with other questionnaires on eating disorders, stress, depression, and anxiety. They completed the hard copy of the EBBS and demographic questionnaire battery within 10 min, and the data were anonymously transferred to Microsoft Excel spreadsheets, and were examined for accuracy and potential data entry errors before the statistical analysis.

### 2.4. Statistical Analysis

Confirmatory factor analysis (CFA) was undertaken using AMOS 23.0 software. Model estimations were based on maximum likelihood methods [42]. In order to establish and assess multi-factor measurement models, we adopted a strategy by Jöreskog (1993), who proposed a model-generating stage before testing the measurement model [43]. First, we assessed one-factor congeneric models for each of the nine EBBS factors. Congeneric models are the smallest entity of a measurement model outlining the regression of a number of observed variables (items) on a single latent variable (factor). This allows re-specification of the model (i.e., freeing covariance, item deletion) before calculating the overall measurement model for exercise benefits and exercise barriers (Aim 1). We then examined the overall fit of the entire EBBS scale (Aim 2). The measurement models were assessed through parsimonious fit, and we employed a chi-square test (χ^2^); the incremental fit was examined through the Comparative Fit Index (CFI) [44] and the Tucker–Lewis Index (TLI) [45], and the absolute fit was analyzed through the standardized root mean square residual (SRMR) [46] and the root means square error of approximation (RMSEA) [47]. Based on the standards developed by Hu and Bentler [48], a very good fit of the data is achieved when CFI and TLI scores exceed 0.95, the SRMR is below 0.08, and the RMSEA is below 0.06. Once the best fitting model was established, we used measurement invariance techniques, as proposed by Gregorich (2006), Byrne (2004; 2010), and Cheung and Rensvold (2002) [49,50,51,52], to assess the covariance structure of the main models across gender (Aim 3).

## 3. Results

### 3.1. Reliability

Cronbach’s alpha showed adequate values for the EBBS (α = 0.82) and the benefits (α = 0.83) and barrier (α = 0.81) subscales. Most subscales showed adequate Cronbach alpha values above the 0.70 threshold [39]. As the benefit subscales ranged between 0.71 and 0.85, the barrier subscales showed some values below the acceptable limit. While exercise milieu (α = 0.73) and time expenditure (α = 0.70) were just above the cutoff value, the internal consistency of the physical exertion factor was marginally below (α = 0.62), and family discouragement showed a particularly low score of 0.47. Given the problematic factor construction that was based on only two items, which may have contributed to the low alpha scores, it was decided to omit the family discouragement factor, excluding items 21 and 33 from further analysis.

### 3.2. Correlations

In Table 2, the correlations matrix revealed good support for the two main constructs of exercise benefits and barriers. All benefit factors showed strong, positive correlations with the benefit measure, ranging between 0.66 and 0.84. The correlation coefficients between the barrier factors and barriers measures also displayed strong and positive links between 0.60 and 0.80. The relationship between benefits and barriers was reflected by a significant, negative coefficient of 0.39.

### 3.3. Convergent Validity

For the exercise benefit higher-order factor, all items showed adequate factor loadings between 0.49 and 0.79. Only one item of the life enhancement factor (item 29, “Exercise helps me decrease fatigue”) showed a slightly lower loading of 0.39. For the barrier factor, items generally varied in factor loadings between 0.46 and 0.77. Item 28 (“I think people in exercise clothes look funny”) revealed a loading of 0.35. Except for physical performance, the congeneric models showed a very good fit of the data in relation to CFI values of 0.95 and above. The TLIs were more variable, with only two factors showing an excellent fit, that is, life enhancement and time expenditure.

Almost all RMSEA values were slightly elevated, i.e., above 0.08 [48]. Life enhancement and exercise milieu showed adequate scores, including the 90% RMSEA, showing promising confidence intervals. Even though all regression weights were significant and fit indices showed some support of the factors’ convergent validity, there were some scores that appeared rather high, and could cause issues in further model testing. Therefore, we examined all models with elevated RMSEAs above 0.08 more closely. The preventive health model revealed a poor absolute fit index, with an RMSEA of 0.133 and a confidence interval ranging between 0.070 and 0.209 (χ^2^ (1) = 10.971, *p* < 0.001; CFI = 0.970; TLI = 0.910; SRMR = 0.035). The RMSEA is a moderately sensitive simple model misspecification, which can occur in congeneric model testing, but the RMSEA is more sensitive to complex model misspecifications [46]. Following up the elevated RMSEA scores, we used the Lagrange Multiplier test for standardized residual covariances to examine model misspecifications. Misspecifications occurred for all three items with strong cross-loadings, i.e., above 10, and error terms strongly covaried, ranging from 14 to 32. Acceptable limits for covariances would be lower than 4 [50]. Therefore, we decided to delete items 5 (“I will present heart attacks by exercising”), 13 (“Exercising will keep me from having high blood pressure”), and 27 (“I will live longer”).

The RMSEA (0.090) for physical performance was also inflated. The Lagrange Multiplier test detected higher scores for the regression weights and error covariances for items 23 (“Exercise improves my flexibility”), 31 (“My physical endurance is improved by exercising”), and 32 (“Exercising improves my self-concept”). Following the deletion of these three items, the remaining six-item factor of physical performance showed adequate scores at this level of model testing (Table 3). Testing psychological outlook, the six-item congeneric model also revealed an elevated RMSEA of 0.106, indicating model misspecifications. The Lagrange Multiplier test for standardized residual covariances indicated cross-loadings above 10 between items 8 and 20. In addition, the error terms for both items substantially co-varied, with M.I. = 25.221. We deleted item 20 (“I have improved feelings of well-being from exercise”) and re-ran the model presented in Table 3. The absolute fit of social interaction was marginally elevated, with an RMSEA of 0.088. Modification indices suggested slightly increased error terms between 4.0 and 5.5. In this instance, we decided to retain all four items of the social interaction factor.

With regard to barriers to exercise, life enhancement showed adequate fit indices, indicating that the data fit the model well. Time expenditure showed a slightly increased RMSEA, but, on closer inspection, all items and covariances indicated appropriate scores. Physical exertion, however, showed some serious model misspecifications, with χ ^2^(1) = 36.908, *p* < 0.001; CFI = 0.831; TLI = 0.494; SRMR = 0.103; RMSEA = 0.252, confidence interval 0.187–0.325. Within the three-item factor, items 19 (“I am fatigued by exercise”) and 40 (“Exercise is hard work for me”) loaded on each other, with M.I. = 33.013 and M.I. = 32.272, respectively, and both error terms co-varied substantially, with M.I. = 31.670. Therefore, we deleted both items from further analysis. Two out of three items measuring physical exertion showed severe misspecifications, and therefore, we omitted this factor from further analysis.

### 3.4. Hierarchical Confirmatory Factor Analysis

When examining multi-group invariance, Byrne [50,51] proposed a testing strategy that involves a number of hierarchical steps. Firstly, a baseline model needs to be established for each sample. Byrne (2010) suggested that testing should be guided by the best fit of the data and model parsimony [50]. In this section, we tested a number of measurement models to establish the best fit. The best-fitting models for the benefit, barrier, or overall EBBS scale were used in further analysis to examine gender differences. Based on the result of the congeneric model testing, we examined a four-factor exercise benefit and three-factor exercise barrier model.

Testing the benefit scale first, the results of the CFA showed a poor fit of the data for the single-factor model, the second-order model, and the four-factor uncorrelated model (Table 3). The four-factor correlated model (M4) showed a slightly better fit, with χ^2^ (164) = 586.433, *p* < 0.001; CFI = 0.914; TLI = 0.903; SRMR = 0.049; and RMSEA = 0.058. The correlated model largely reflected the original five-factor model as proposed by Sechrist et al. [28], and showed the best fit of the data, which was therefore used for further analyses (Table 4).

A number of items in the four-factor solution appeared to be problematic. Cross-loading items and modification indices for regression weights with scores above 10 and covariances above 4 (Lagrange Multiplier test for standardized residual covariances) indicated poor fit of three items. Two life enhancement items and one psychological outlook item were detected as causing misspecifications. Item 8 (“Exercise gives me a sense of personal accomplishment”) contributed largely to the misspecification of the model, as it cross-loaded on physical performance (M.I. = 10.831) and other items (M.I. = 37.146), and showed large error covariances with other factors (physical performance, M.I. = 42.381) and items (e.g., item 15, M.I. = 33.966). Following the deletion of this item, the amended model showed a better fit (M5). Similarly, strong impacts on the model fit were found for item 36 (“Exercise improves the quality of my work”) and item 25 (“My disposition is improved with exercise”). Deletion of both life enhancement items resulted in a much better model fit (M6), which, according to Hu and Bentler (1999) [48], almost reached excellent fit (M7).

Examining the barrier scale, the results of the CFA showed a poor fit for the single-factor and the uncorrelated two-factor models, whereas a better fit was found for the second-order model and the two-factor correlated model (M4). The Lagrange Multiplier test showed severe misspecification in association with item 9, including cross-loadings (M.I. = 18.260) and error covariances (M.I. = 17.903). Deletion of item 9 (“Places for me to exercise are too far away”) improved the model to a good and excellent fit (M5), with χ^2^ (19) = 61.855, *p* < 0.001; CFI = 0.953; TLI = 0.931; SRMR = 0.044; and RMSEA = 0.063.

A number of items in the four-factor solution appeared to be problematic. Cross-loading items and modification indices for regression weights with scores above 10 and covariances above 4 (Lagrange Multiplier test for standardized residual covariances) indicated poor fit of three items. Two life enhancement items and one psychological outlook item were detected as causing misspecifications. Item 8 (“Exercise gives me a sense of personal accomplishment”) contributed largely to the misspecification of the model, as it cross-loaded on physical performance (M.I. = 10.831) and other items (M.I. = 37.146), and showed large error covariances with other factors (physical performance, M.I. = 42.381) and items (e.g., item 15, M.I. = 33.966). Following the deletion of this item, the amended model showed better fit (M5). Similarly, strong impacts on the model fit were found for item 36 (“Exercise improves the quality of my work”) and item 25 (“My disposition is improved with exercise”). Deletion of both life enhancement items resulted in a much better model fit (M6), which, according to Hu and Bentler (1999) [48], almost reached excellent fit (M7).

Examining the barrier scale, the results of the CFA showed a poor fit for the single-factor and the uncorrelated two-factor model, whereas better fit was found for the second-order model and the two-factor correlated model (M4). The Lagrange Multiplier test showed severe misspecification in association with item 9, including cross-loadings (M.I. = 18.260) and error co-variances (M.I. = 17.903). Deletion of item 9 (“Places for me to exercise are too far away”) improved the model to good and excellent fit (M5), with χ^2^ (19) = 61.855, *p* < 0.001; CFI = 0.953; TLI = 0.931; SRMR = 0.044; and RMSEA = 0.063.

Finally, we merged the benefit and barrier models into a six-factor model consisting of 27 items (Table 4). The CFA results indicated modest values for the EBBS measurement model, M1. One item cross-loaded on various factors, and was subsequently deleted. The item was part of the exercise milieu factor (item 12 “I am too embarrassed to exercise”), and loaded on to life enhancement (M.I. = 10.138), social interaction (M.I. = 24.470), and psychological outlook (M.I. = 21.806). The retested model, M2, substantially increased in the model fit. In addition, two error covariances have been found with overlapping content, i.e., items 2 (“Exercise decreases feelings of stress and tension for me”) and 3 (“Exercise improves my mental health”). Hierarchical CFA testing allowed us to re-specify the benefit, barrier, and EBBS measurement models. With regard to the second aim, it appears that the benefit and barrier models should be preferred for further testing, as fit indices showed stronger values for the four- and two-factor models. Items that were retained following the hierarchical CFA and factor loadings are presented in Table 5 and Table 6.

### 3.5. Gender Invariance

For multi-group invariance testing, Gregorich (2006) proposed a hierarchical approach to the testing procedure, including the comparison of three models assessing metric invariance (factor loadings constrained to be invariant), strong invariance (factor loadings and item intercepts constrained to be invariant), and strict invariance (factor loadings, item intercepts, and item variance) against a configural model that is freely estimated with no constraints [49]. Support for metric invariance would indicate whether factors have the same meaning across national samples, strong invariance indicates whether comparisons between latent mean differences will be meaningful, and strict invariance takes into consideration item residual invariance, providing an even stronger basis for cross-cultural comparisons [49].

In Table 7, the results between configural and metric models showed acceptable values for all group comparisons, with ΔCFI < 0.01 [52]. Gregorich (2006) stated that this comparison is particularly important, as it indicates whether items have the meaning between groups [49]. Testing the barriers model, the results indicated that all items had meaning across gender groups (M2).

Testing for strong invariance by constraining factor loadings and item intercepts of the model, in accordance with Cheung and Rensvold’s (2002) suggestions [52], ΔCFI < 0.01, the results showed strong support for the model comparison between male and female data, with ΔCFI = 0.008. Gregorich (2006) proposed that the equivalence of factor item loadings and item intercepts is a precondition for testing latent mean differences [49], as applied by Vlachopoulos et al. (2013) [53]. The current results indicated that it would be appropriate to proceed with this type of analysis to compare the mean differences between males and females in their perception of exercise barriers. Finally, strict invariance was not evident for the two gender models, with ΔCFI = 0.012. The test of mean difference showed no significant results.

In Table 8, the results of exercise benefits between configural and metric models also showed acceptable values for all group comparisons, with ΔCFI < 0.01 [52], although the results of M2 indicated a significant difference between the configural and metric models, with *p* < 0.05. A shortcoming of this technique is that it only allows examination by comparison of all factor loadings across groups, but does not provide pinpoint accuracy when it comes to comparing items for specific subscales across groups. Although Cheung and Rensvold (2002) stated that the measurement model is completely invariant to the configural model when ΔCFI is less than 0.01 [52], Byrne (2010) considered Δχ^2^ and Δdf in conjunction with statistical significance as more stringent [50]. More importantly, in order to show that non-invariance testing proceeds on a subscale level, this way, the results allow to pinpoint which items are invariant and non-invariant across groups [51]. With regard to the data, the comparison between data from each gender did not show substantial differences on a factor-loading level, with ΔCFI = 0.003, indicating that factor loadings were invariant across gender groups, whereas the *p*-value, *p* < 0.05, indicated a significant difference between the models. This may suggest that specific items have different meanings, or are understood differently between males and females.

Following the test of the metric model, which had all factor loadings constrained, Byrne (2010) suggested to constrain the items of the first latent variable, in this case, items of the barriers factor equal across groups, and to measure the remaining items freely for each sample [50]. Therefore, when “factor-loading parameters are found to be invariant across groups, their specified equality constraints are maintained, cumulatively, throughout the remainder of the invariance-testing process” (p. 223). This procedure is shown in Table 8 (see Models 3 to 6). When items for each subscale were constrained separately, all subscale items for life enhancement, physical performance, and social interaction performed well and supported invariance across groups. For the psychological outlook factors, one item had to be estimated freely (“I enjoy exercise”), indicating significant differences in item meaning between groups. Model M7 showed no substantial differences regarding ΔCFI = 0.001. The following test of mean differences was not significant.

## 4. Discussion

The overall purpose of the current study was to investigate the reliability and validity of the EBBS questionnaire and to examine whether the EBBS is invariantly perceived across gender. To the best of our knowledge, this is the first and most detailed and extensive cross-gender investigation of the reliability and validity of the EBBS that has uniquely and systematically conducted a CFA in addition to the previous tests and examined the perceived gender invariance of the EBBS factors and items. Our findings partly confirm the results of the previous EFA of the EBBS, which identified a core of factors and items within the EBBS that are relevant and essential to the validity and reliability of the questionnaire across populations.

The first aim of the study was to examine the reliability of the EBBS on a factor and global level. The results showed strong evidence for the internal consistency for global measures of benefits and barriers, which supports research findings by Sechrest et al. [28]. Some Cronbach alpha scores were below the desirable 0.70 cutoff [39], namely physical exertion and family discouragement. The two-item factor of family discouragement showed a particularly low alpha value of 0.46, and was omitted from further analysis. Previous research also indicated variability in alpha scores, as reported by Akbari Kamrani et al. [29], for psychological outlook (0.58), exercise milieu (0.65), and time expenditure (0.60). These findings bear similarity with previous studies that also found reliability issues on a subscale level (e.g., Brown [30]; Ortabag et al. [31]).

The second aim of this study was to investigate the latent factor structure of the EBBS. Following rigorous assessments on an item level, i.e., congeneric model testing and first- and second-order level, i.e., hierarchical CFA testing, the results provided evidence for a four-factor structure of the benefits measure, namely life enhancement, physical performance, psychological outlook, and social interaction, and a two-factor structure of the barriers measures, including exercise milieu and time expenditure. The current findings largely support the initial EBBS nine-factor structure proposed by Sechrest and colleagues [28]. Following the validation stage, Akbari Kamrani et al. [29] and Ortabag et al. [31] extracted five of the six factors found in this study through exploratory factor analyses. Based on the evidence provided in this and previous studies, there appears to be a core of factors consistently supporting the measurement of exercise barriers and benefits.

Factors that have not been supported in the present and previous studies might lack relevance, i.e., preventive health, physical exertion, or psychometric soundness, i.e., family discouragement. For instance, family discouragement showed a weak alpha value of 0.46 (Table 2), and consisted of only two items, making it prone to estimation problems [54]. Kline (2005) proposed that any measurement factor should consist of at least three indicators [54], and this suggestion has been taken into consideration by Brown [30], who omitted family discouragement due to this violation. Furthermore, we found no support for the physical exertion and preventive health factors at the congeneric model testing level, that is, all three items of each factor showed severe misspecification, and were omitted from further analysis. This was due to a particularly high RMSEA, which is sensitive to the number of items tested and favors model parsimony [55]. The results showed that the chosen item estimates did not fit the covariance matrix [56]. Preventive health did not emerge as a relevant factor in previous research (e.g., Akbari Kamrani et al. [29]), as well as time expenditure (e.g., Brown, [30]; Ortabag et al. [31]). It could be argued that preventive health is a rather broad factor, and participants, particularly younger age groups, such as student samples, may not be concerned with health issues, such as high blood pressure or heart attacks, that may or may not occur in the distant future.

The correlation between global measures of benefits and barriers was significant at r = −0.39. This finding is similar to Brown [30], with r = −0.46. On a subscale level, we generally found moderate to strong correlations (Table 2). Brown [30] highlighted that measures of benefits and barriers bear importance for the EBBS, but he advised against the separation of both measures. In the current study, the hierarchical model testing confirmed sound psychometric properties and excellent indices for the correlated four-factor benefits model, the correlated two-factor barriers model, and the correlated six-factor model (Table 4). These results indicate that future research could use measures of exercise barriers and exercise benefits either separately or in conjunction, depending on the specific aims of the study.

The original EBBS by Sechrest et al. [28] incorporated 43 items, whereas the current 26 items strongly supported the six-factor measure. The current model indicates parsimony, and the factor loadings for the retained items (Table 5 and Table 6) showed convergent validity, with loadings generally above 0.45. Tabachnick and Fidell [57] argued that item loadings below 0.45 can cause problems in factor structure interpretation, as they would explain less than 20% of the variance. One item of the life enhancement factor (“Exercise helps me decrease fatigue”) showed a loading of 0.35. Despite the recommendations by Tabachnick and Fidell [57], we decided to retain this item because of the overall performance of the life enhancement factor was strong across congeneric and hierarchical model testing.

The third aim of the study was to examine the potential gender differences between exercise factors and item understanding using multi-group confirmatory analysis. This type of assessment is relevant to make appropriate cross-cultural group comparisons [52]. When all factor loadings for the benefits and barriers subscales were constrained to be equal, the results showed no significant differences between male and female participants. Out of the 26 items, only one item, “I enjoy exercise” (life enhancement factor), had to be measured freely, indicating that male and females have a different understanding of what exercise enjoyment means. Although some support for metric invariance has been found, strong invariance at an intercept level could not be confirmed, and no latent mean differences were found. The results generally support the notion that the EBBS, more specifically the six-factor version tested in this study, is equally valid to use with male and female participants.

The results of this study add the following information to the extant literature. The psychometric properties of the amended, parsimonious EBBS are sound, providing support for its reliability and validity. Methodologically, the current study goes beyond previous EFA testing, and incorporates a rigid systematic protocol for testing the latent factor structure of the EBBS, consisting of first-order factor analysis (i.e., congeneric models), second-order factor analysis (i.e., CFA of the benefits and barriers model, and for the full EBBS model), and multi-group CFA to examine gender differences in the use and understanding of the EBBS. The use of this protocol was based on contentions by key references on model testing, including Byrne [50], Cheung and Rensvold [52], Gregorich [49], and Hu and Bentler [46,48]. Previous research on EBBS model testing did not follow such a systematic test protocol, which appears to be a methodological shortcoming. Future research testing the psychometric properties of the EBBS should follow clear guidelines on the testing procedures.

The current study was conducted within the framework of a large cross-sectional study called the Collaborative Investigation in Nutritional Status of Young Adults (CINSYA), which recruited a large sample of participants via convenient sampling, mainly from young adults studying in higher education institutions in Merseyside, England [40,41]. Ideally, the investigation of the reliability, validity, and gender invariance as seen within the current study would require a large but heterogeneous sample to be representative of the broad target population, and we feel that the rather narrow age range (mostly 18–25 years) represented in the current study may limit the generalizability of our results. Therefore, we believe that our findings should be considered in view of the above limitation. A methodological weakness that should be addressed is the use of calibration and validation samples [43]. Ideally, two independent samples should be used, including a calibration sample, to generate an initial structure and establish its factor structure, followed by the assessment of a validation sample to confirm this initial structure [43]. In this study, the latent factor structure was based on Sechrest et al. [28], who incorporated a large sample of 664 participants in their EFA test procedure. Researchers should consider the advantages of a stronger methodological approach, such as the inclusion of two samples. This approach could be used in cross-cultural studies trying to establish the cross-cultural validity of the EBBS. This would be helpful, as previous research has been conducted with samples from Iran (Akbari Kamrani et al. [29]), Turkey (Ortabag et al. [31]), and China (Guo [58]), but so far only Guo [58] implemented EFA and CFA analyses, although s/he did not use two independent samples.

The focus of the current study was on the reliability, validity, and gender invariance, and not the actual investigation of the benefits and barriers of PA amongst the target population. Despite this, we decided to collate a summary of the previous studies across the world to facilitate the comparison of our subscale means of the perceived benefits and barriers of PA against the previous studies with comparable methodologies and populations (Table 9). While most mean perceived benefit subscales seem to be generally in line with the mean subscales reported elsewhere, the mean perceived barriers of PA (e.g., in family discouragement, exercise milieu, and time expenditure) within the current study are not consistent with the other scores previously reported. Such cross-cultural inconsistencies in the perceived subscales are not unexpected, however, we consider these in view of our findings, and argue that these inconsistencies might partially be the reflection of the rigor of a useful research instrument that would need further investigation, revision, and cross-cultural validation.

## 5. Conclusions

The psychometric properties of the EBBS provide support for a reliable and valid operationalization of exercise benefits and exercise barriers. The results indicated that, in comparison with the original validation of the EBBS by Sechrest and colleagues [28], a number of core factors and items are still relevant for the assessment of higher-order factors. The current 26-item version in contrast to the Sechrest et al.’s [28] 43-item version displays a better parsimony, although, in agreement with Brown [30], the number of barrier factors could be increased to avoid under-powering of future assessments.

The current study contributes to the literature in operational and conceptual levels. While previous factor analysis including the studies of Akbari Kamrani et al. [29], Brown [30], and Sechrist et al. [28] used exploratory factor analysis, the current study replicated the factor structure based on the previous evidence through a statistically rigorous approach using congeneric model testing and confirmatory factor analysis, including hierarchical models and gender invariance testing. Our findings indicated strong support for the scale’s internal consistency. In contrast to the previous studies, proposing a 10-factor structure of the EBBS, our CFA-based findings could only confirm a 6-factor structure containing two factors associated with the perception of barriers, and four to perception of benefits. The remaining four factors revealed substantial issues on an operational level, and future studies need to wary when or if using the factors in questions. If all EBBS factors are to be applied in the future use of the instrument, the rigor of the study can be improved by conducting additional factor analysis as part of the study, which may potentially lead to omitting the factors that are underperforming on a measurement level. Our study is an exemplar in which not all items performed well due to limited reliability and cross-loading and lack of gender invariance. Researchers using EBBS cross-culturally need to be aware of these shortcomings in preparation, design, and implementation of their studies.

Future research needs to validate additional content areas of exercise barriers, as the current and previous findings (e.g., Brown [30]) suggested an imbalance between benefits, i.e., four or five factors, and barriers, i.e., two factors. Albeit the results of the present and previous studies (e.g., Akbari Kamrani et al. [29]; Brown. [30]; Ortabag et al. [31]) indicated a number of core barriers, namely exercise milieu and time expenditure, and additional barriers that were formed in previous EFAs. The results of this study also underlined the value of two global categories (exercise benefits and exercise barriers) for future research as relevant constructs that could be used on its own or in conjunction.

## Figures and Tables

**Table 1:** Summary of the literature on the perception of the benefits and barriers of physical activity with methodology and population comparable to the current study.

	Author/s and Date	Population	Methods Summary	Key Findings	Strengths	Limitations
1	Fredrick et al. (2020) Reference [34]	862 university students, USA	EBBS questionnaire Resistance training questionnaire International Physical Activity Questionnaire (IPAQ)	PP: 3.5 ± 0.4, PO: 3.4 ± 0.5, PH: 3.3 ± 0.5, LE: 3.2 ± 0.5, SI: 2.7 ± 0.6 PE: 2.7 ± 0.6, TE: 1.8 ± 0.5, EM: 1.6 ± 0.5, FD: 1.5 ± 0.5 - Males reported more perceived benefits to EX/PA - No significant interaction effects of gender and year of study in behaviors or perceived benefits and barriers	- Investigation of the correlation between EX behavior and barriers and benefits of EX/PA - Large sample size and relatively proportionate representation of year groups in the sample	- 96% met aerobic PA guidelines (participation bias?) - Possible impact of availability of university/community bus system - Unequal gender and racial/ethnic make-up of the sample - Potential impact of seasonality, temperature, and geographical location on PA/EX behavior and perceptions
2	Firdaus Abdullah et al. (2018) Reference [11]	355 university students, Malaysia	Demographic & EBBS questionnaires	PP: 4.18 ± 0.56, PO: 4.16 ± 0.59, PH: 3.89 ± 0.63, LE: 3.97 ± 0.54, SI: 3.96 ± 0.67 PE: 2.96 ± 0.74, TE: 2.37 ± 0.69, EM: 2.60 ± 0.64, FD: 2.28 ± 0.87	- Reasonable sample size - Attention to characterization of demographics	- Inadequate reporting of total scales of barriers and benefits - Lack of a tool to assess PA/EX behavior - Poor description of methods and data analysis
3	Lovel et al. (2010) Reference [6]	200 non-exercising female university students, UK	Brief demographic questionnaire EBBS questionnaire	PP: 3.25 ± 0.46, PO: 3.08 ± 0.60, PH: 3.05 ± 0.56, LE: 2.93 ± 0.48, SI: 2.50 ± 0.65 PE: 2.63 ± 0.60, TE: 2.12 ± 0.59, EM: 2.08 ± 0.60, FD: 2.06 ± 0.62 - Significantly higher perceived benefits vs. barriers - PP rated significantly higher than other benefits and PE rated significantly higher than other barriers	- Data collected via random selection of participants from two different universities on three different occasions	- Focus on non-exercising female population restricted external validity - Inadequate demographic assessment, including lack of data on ethnicity, year of study, family care responsibilities, wider socioeconomic characteristics, or other confounding variables
4	Szarabajko (2018) Reference [35]	595 overweight, obese, and normal weight university students, USA	EBBS questionnaire Body composition through bioelectrical impedance body fat analyzer, Waist circumference	PP: 3.46 ± 0.42, PO: 3.24 ± 0.50, PH: 3.46 ± 0.45, LE: 3.31 ± 0.45, SI: 3.16 ± 0.54 PE: 2.12 ± 0.57, TE: 3.00 ± 0.57, EM: 3.20 ± 0.52, FD: 3.25 ± 0.64 - Obese students demonstrated greater perceived barriers, while normal weight students reported greater benefits to exercise. - Normal weight students reported higher perceived benefits for LH, but not for PH.	- Use of body composition and waist circumference together with the EBBS - First study to link waist circumference and the EBBS - Strong exclusion criteria leading to enhancing the rigor - Large sample size	- Some barriers constructed might need further revision and rephrasing for the population per the low alpha reported. - Errors arising from self-reported anthropometry - Potential confounding impact of health knowledge and education on reported findings
5	Nolan et al. (2011) Reference [36]	462 first-year university students, South Africa	EBBS questionnaire	PP: 3.22 ± 0.43, PO: 3.16 ± 0.44, PH: 3.23 ± 0.55, LE: 3.02 ± 0.39, SI: 2.76 ± 0.62 PE: 2.28 ± 0.56, TE: 1.93 ± 0.60, EM: 2.05 ± 0.46, FD: 1.90 ± 0.70 - Higher scores of PP for males vs. females - The mean scores for male students were slightly higher for PE and EM as barriers, whereas the mean scores for female students were higher for TE and family FD as barriers.	- Analysis of gender difference in the perception of benefits and barriers of exercise	- Purposive sampling - Inadequate demographics and limited external validity because of the focus on freshman students
6	Dalibalta & Davison (2016) Reference [37]	100 university students, UAE	EBBS questionnaire Godin 1997 leisure time exercise questionnaire	PP: 3.39 ± 0.10, PO: 3.17 ± 0.22, PH: 3.26 ± 0.11, LE: 3.04 ± 0.16, SI: 2.59 ± 0.22 PE: 2.67 ± 0.17, TE: 2.22 ± 0.38, EM: 1.88 ± 0.32, FD: 1.87 ± 0.01 - 35% of participants never/rarely exercised, while 44% exercised only sometimes	- Random selection - Ethnic diversity - First study of the perceived benefits and barriers of PA/EX in this population	- Relatively small sample size - Disproportionate gender (81% females), body composition (80% normal weight), and ethnicity - Limited rigor (lack of representative sample and lack of cross-cultural validation of tools) - Inadequate confounding factor control (e.g., climate and culture)
7	Gad et al. (2018) Reference [38]	400 female university students, Saudi Arabia	EBBS questionnaire, Godin 1997 leisure time exercise questionnaire Demographic questionnaire	PP: 3.34 ± 0.556, PO: 3.35 ± 0.553, PH: 3.23 ± 0.642, LE: 3.30 ± 0.558, SI: 3.12 ± 0.658 PE: 2.63 ± 0.730, TE: 2.80 ± 0.724, EM: 2.70 ± 0.639, FD: 2.55 ± 0.867 - 65% of participants physically inactive - Physically inactive students had significantly higher average barriers score compared with active students - Overweight and obese students had significantly higher barriers scores vs. normal weight students	- Simple random selection - Reasonable sample size per the power calculation	- Lack of cross validation of questionnaires - Potential confounding impact of health knowledge and education on reported findings

Note: PP = physical performance, PO = psychological outlook, PH = preventive health, LE = life enhancement, PE = physical exertion, TE = time expenditure, EM = exercise milieu, FD = family discouragement; EX = exercise, PA = physical activity.

**Table 2:** The correlation matrix, Cronbach’s alpha, and descriptive statistics.

		Benefit Factors							Barrier Factors			
		1	2	3	4	5	6	7	8	9	10	11
1.	Benefits (total score)											
2.	Barriers (total score)	−0.39 **										
Benefits												
3.	LE	0.84 **	−0.36 **									
4.	PP	0.84 **	−0.337 **	0.69 **								
5.	PO	0.84 **	−0.38 **	0.63 **	0.68 **							
6.	SI	0.77 **	−0.34 **	0.56 **	0.50 **	0.62 **						
7.	PH	0.66 **	−0.13 **	0.48 **	0.52 **	0.36 **	0.26 **					
Barriers												
8.	EM	−0.40 **	0.71 **	−0.34 **	−0.38 **	−0.42 **	−0.31 **	−0.15 **				
9.	TE	−0.31 **	0.80 **	−0.30 **	−0.29 **	−0.28 **	−0.24 **	−0.13 **	0.51 **			
10.	PE	−0.14 **	0.60 **	−0.18 **	−0.05	−0.18 **	−0.18 **	0.03	0.32 **	0.29 **		
11.	FD	−0.27 **	0.73 **	−0.23 **	−0.23 **	−0.25 **	−0.25 **	−0.11 **	0.30 **	0.47 **	0.16 **	
	Cronbach’s Alpha	0.93	0.81	0.78	0.85	0.83	0.74	0.71	0.73	0.70	0.62	0.46
	Minimum	1	1	1	1	1	1	1	1	1	1	1
	Maximum	4	3.29	4	4	4	4	4	3.33	4	4	4
	M	3.07	2.12	2.98	3.30	3.23	2.77	3.05	1.86	2.00	2.70	1.92
	SD	0.40	0.41	0.45	0.41	0.53	0.61	0.53	0.49	0.58	0.54	0.67

Note. ** Correlations are significant at *p* < 0.01. LE = life enhancement, PP = physical performance, PO = psychological outlook, SI = social interaction, PH = preventive health; EM = exercise milieu, TE = time expenditure, PE = physical exertion, FD = family discouragement.

**Table 3:** Congeneric model testing.

	χ^2^	*p*<	CFI	TLI	SRMR	RMSEA	RMSEA 90% CI
	Benefits						
LE (seven-item factor)	35.883	0.001	0.975	0.963	0.031	0.053	0.032–0.074
PP (nine-item factor)	150.294	0.001	0.923	0.897	0.048	0.090	0.076–0.104
PP (six-item factor)	27.259	0.01	0.981	0.969	0.027	0.060	0.035–0.086
PO (six-item factor)	65.869	0.001	0.951	0.918	0.043	0.106	0.083–0.131
PO (five-item factor)	18.607	0.01	0.985	0.970	0.026	0.069	0.038–0.104
SI (four-item factor)	10.784	0.01	0.981	0.943	0.026	0.088	0.042–0.143
	Barriers						
EM (six-item factor)	27.924	0.01	0.967	0.945	0.033	0.061	0.036–0.087
TE (three-item factor)	5.281	0.05	0.987	0.962	0.020	0.087	0.027–0.166

Note. CFI = Comparative Fit Index; TLI = Tucker−Lewis Index; SRMR = standardized root mean squared residual; RMSEA = root mean square error of approximation; CI = confidence interval; LE = life enhancement; PP = physical performance; PO = psychological outlook; SI = social interaction; EM = exercise milieu; TE = time expenditure.

**Table 4:** CFA of the benefit scale, barrier scale, and EBBS scale.

	χ^2^	*p*<	CFI	TLI	SRMR	RMSEA	RMSEA 90% CI
	Benefits (22 items reduced to 19)						
M1: Single-factor model	1109.662	0.001	0.799	0.778	0.068	0.087	0.082–0.093
M2: Second-order model	631.578	0.001	0.905	0.893	0.054	0.061	0.055–0.066
M3: Uncorrelated four-factor model	1520.435	0.001	0.708	0.677	0.261	0.105	0.101–0.110
M4: Correlated four-factor model	586.433	0.001	0.914	0.903	0.049	0.058	0.052–0.063
M5: Correlated four-factor model (w/o item 8)	490.714	0.001	0.927	0.916	0.046	0.055	0.049–0.060
M6: Correlated four-factor model (w/o item 36)	411.048	0.001	0.936	0.926	0.042	0.052	0.045–0.058
M7: Correlated four-factor model (w/o item 25)	358.718	0.001	0.943	0.933	0.041	0.051	0.044–0.057
	Barriers (9 items reduced to 8)						
M1: Single-factor model	208.444	0.001	0.837	0.783	0.065	0.109	0.096–0.123
M2: Second-order model	100.461	0.001	0.933	0.908	0.049	0.071	0.057–0.086
M3: Uncorrelated two-factor model	251.458	0.001	0.799	0.732	0.166	0.121	0.108–0.135
M4: Two-factor model	100.461	0.001	0.933	0.908	0.049	0.071	0.057–0.086
M5: Two-factor model (w/o item 9)	61.855	0.001	0.953	0.931	0.044	0.063	0.046–0.081
	EBBS (27 items reduced to 26)						
M1: Six-factor model	727.682	0.001	0.915	0.904	0.049	0.049	0.044–0.054
M2: Six-factor model (item 12 deleted)	642.614	0.001	0.924	0.913	0.044	0.047	0.042–0.052
M3: Six-factor model (e2–e3)	613.675	0.001	0.930	0.919	0.044	0.046	0.041–0.050

Note. CFI = Comparative Fit Index; TLI = Tucker−Lewis Index; SRMR = standardized root mean squared residual; RMSEA = root mean square error of approximation; CI = confidence interval.

**Table 5:** Benefits—retained items and factor loadings.

Factors and Items	Item Loadings
LE—Exercising helps me sleep better at night.	0.58
LE—Exercise helps me decrease fatigue.	0.35
LE—Exercising increases my mental alertness.	0.67
LE—Exercise allows me to carry out normal activities without becoming tired.	0.68
LE—Exercise improves overall body functioning for me.	0.62
PP—Exercise increases my muscle strength.	0.62
PP—Exercise increases my level of physical fitness.	0.68
PP—My muscle tone is improved with exercise.	0.69
PP—Exercising improves functioning of my cardiovascular system.	0.72
PP—Exercise increases my stamina.	0.68
PP—Exercise improves the way my body looks.	0.55
PO—I enjoy exercise.	0.77
PO—Exercise decreases feelings of stress and tension for me.	0.72
PO—Exercise improves my mental health.	0.66
PO—Exercising makes me feel relaxed.	0.64
SI—Exercising lets me have contact with friends and persons I enjoy.	0.67
SI—Exercising is a good way for me to meet new people.	0.64
SI—Exercise is good entertainment for me.	0.73
SI—Exercising increases my acceptance by others.	0.51

Note. LE = life enhancement, PP = physical performance, PO = psychological outlook, SI = social interaction.

**Table 6:** Barriers—retained items and factor loadings.

Factors and Items	Item Loadings
EM—It costs too much to exercise.	0.55
EM—Exercise facilities do not have convenient schedules for me.	0.62
EM—I think people in exercise clothes look funny.	0.43
EM– There are too few places for me to exercise.	0.60
TE—Exercising takes too much of my time.	0.53
TE—Exercise takes too much time from family relationships.	0.73
TE—Exercise takes too much time from my family responsibilities.	0.76

Note. EM = exercise milieu, TE = time expenditure.

**Table 7:** Summary of fit statistics for tests of gender invariance—barriers.

Model	Model Comparison	χ^2^	*df*	Δχ^2^	Δ*df*	Statistical Significance	CFI	ΔCFI
M1: Configural model (no equality constraints—seven items)	-	69.314	26	-	-	-	0.944	-
M2: All item factor loadings constrained ^a^	2 vs. 1	76.311	33	6.997	7	NS	0.944	0.000
M3: Factor loadings and item variances constrained	3 vs. 1	89.963	40	20.649	14	NS	0.936	0.008
M4: Factor loadings, item variances, and covariances constrained	4 vs. 1	93.821	41	24.507	15	NS	0.932	0.012

Note. ^a^ Kline (2005) proposed that items which are fixed to 1.0 cannot be examined for invariance. Therefore, these items were freed, and the latent parent variables were fixed to 1.0. NS = not significant.

**Table 8:** Summary of fit statistics for tests of gender invariance—benefits.

Model	Model Comparison	χ^2^	*df*	Δχ^2^	Δ*df*	Statistical Significance	CFI	ΔCFI
M1: Configural model (no equality constraints—19 items)	-	540.040	292				0.932	-
M2: All item factor loadings constrained ^a^	2 vs. 1	571.204	311	31.164	19	*p* < 0.05	0.929	0.003
M3: Items for LE constrained	3 vs. 1	546.096	297	6.056	5	NS	0.932	0.000
M4: Items for LE and PP constrained	4 vs. 1	548.363	303	8.323	11	NS	0.933	0.001
M5: Items for LE, PP, and PO constrained	5 vs. 1	551.636	307	11.596	15	NS	0.933	0.001
M6: Items for LE, PP, PO, and SI constrained (item 1 freely estimated)	6 vs. 1	562.899	310	22.859	18	NS	0.931	0.001
M7: Factor loadings and item variances constrained	7 vs. 1	609.870	329	69.830	37	*p* < 0.01	0.923	0.009
M8: Factor loadings, item variances, and covariances constrained	8 vs. 1	625.807	335	85.767	43	*p* < 0.001	0.920	0.012

Note. ^a^ Kline (2005) proposed that items which are fixed to 1.0 cannot be examined for invariance. Therefore, these items were freed and the latent parent variables were fixed to 1.0. LE = life enhancement; PP = physical performance; PO = psychological outlook; SI = social interaction; NS = not significant. Item 1, “I enjoy exercise”, of the psychological outlook factor had to be estimated freely.

**Table 9:** Comparison of the subscale means of perceived benefits and barriers of the physical activity as reported in the current study compared with the previous studies with comparable methodology and population.

Study Results for EBBS Factors	Lovel et al. (2010) UK	Nolan et al. (2011) South Africa	Dalibalta & Davison (2016) UAE	Gad et al. (2018) Saudi Arabia	Szarabajko (2018) USA	Firdaus Abdullah et al. (2018) Malaysia	Fredrick et al. (2020) USA	The Current Study (2021) UK
Reference	[6]	[36]	[37]	[38]	[35]	[11]	[34]	-
Benefit Subscale								
Physical Performance	3.25	3.22	3.39	3.34	3.46	4.18	3.5	3.30
Psychological Outlook	3.08	3.16	3.17	3.35	3.24	4.16	3.4	3.23
Preventive Health	3.05	3.23	3.26	3.23	3.46	3.89	3.3	3.05
Life Enhancement	2.93	3.02	3.04	3.30	3.31	3.97	3.2	2.98
Social Interaction	3.50	2.76	2.59	3.12	3.16	3.96	2.7	2.77
Barriers Subscale								
Physical Exertion	2.63	2.28	2.67	2.63	2.12	2.96	2.7	2.30
Time Expenditure	2.12	1.93	2.22	2.80	3.00	2.37	1.8	3.00
Exercise Milieu	2.08	2.05	1.88	2.70	3.20	2.60	1.6	3.14
Family Discouragement	2.06	1.90	1.87	2.55	3.25	2.28	1.5	3.08

## Data Availability

Data are available to reviewers upon request.
